# Supplementary material for: The creative interplay between hand gestures, convergent thinking, and mental imagery
Source: PLoS One. 2023 Apr 6;18(4):e0283859. doi: 10.1371/journal.pone.0283859 (PMC10079032; doi:10.1371/journal.pone.0283859)
Supplement: S1 File — A file including a detailed description and visualisation of gesture types and frequencies across groups and conditions and the linear mixed-effects modelling results for the individual representational gesture types, i.e., iconic, metaphoric and pointing/deictic gestures, and palm-revealing gestures. (DOCX) [file pone.0283859.s001.docx]

**Supporting Information**

***Gesture Use***

Gesture use frequency increased significantly during the RAT in the gesture-encouraged condition of Group 1 (GE1), *t*(38) = - 4.62, *p* < .001. There was no significant difference between the gesture use frequency during the RAT of the gesture-encouraged conditions of Group 1 (GE1) and Group 2 (GE2), *t*(76) = 1.91, *p* = .06 (see Figure S1).

For the vRAT, there was also an increase in gesture use frequency in the gesture-encouraged condition of Group 1 (GE1), *t*(39) = - 4.57, *p* < .001, and no difference between the gesture-encouraged conditions of Group 1 (GE1) and Group 2 (GE2), *t*(64) = 1.64, *p* = .106 (see Figure S2). Distribution of gesture types across groups and conditions for the RAT and the vRAT are presented in Figure S3 and Figure S4.


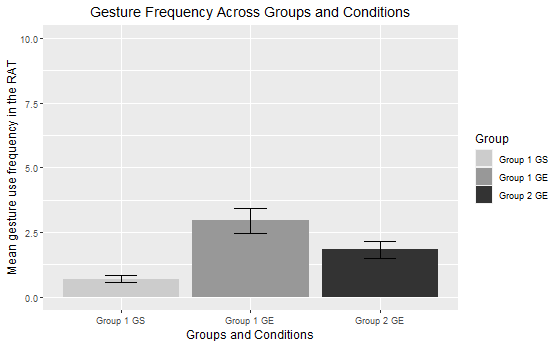


**Figure S1.** Mean gesture use frequency during the RAT for both conditions (gesture-spontaneous (GS) and gesture-encouraged (GE)) and both groups (Group 1 and Group 2). Gesture frequency for each item was calculated by dividing the total number of gestures by the total word count.


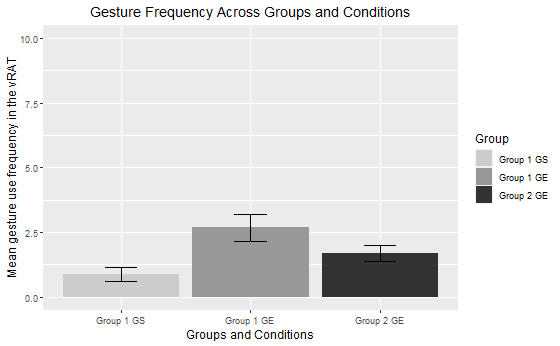


**Figure S2.** Mean gesture use frequency during the vRAT for both conditions (gesture-spontaneous (GS) and gesture-encouraged (GE)) and both groups (Group 1 and Group 2). Gesture frequency for each item was calculated by dividing the total number of gestures by the total word count.

**
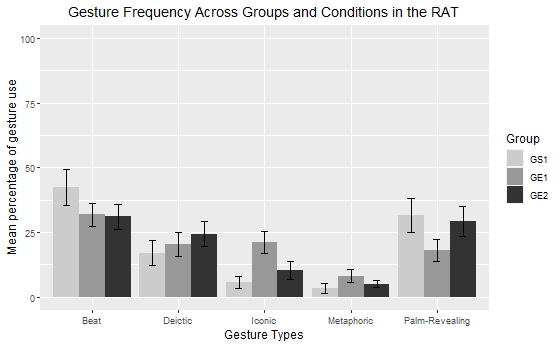
**

**Figure S3.** Mean percentage of gesture use frequency across gesture types during the RAT for both conditions (gesture-spontaneous (GS) and gesture-encouraged (GE)) and both groups (Group 1 and Group 2). Gesture frequency for each item was calculated by dividing the total number of gestures by the total word count.


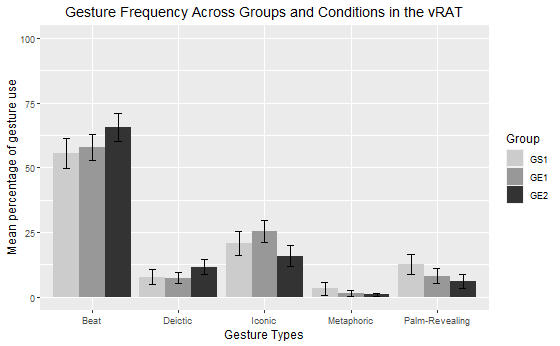


**Figure S4.** Mean percentage of gesture use frequency across gesture types during the vRAT for both conditions (gesture-spontaneous (GS) and gesture-encouraged (GE)) and both groups (Group 1 and Group 2). Gesture frequency for each item was calculated by dividing the total number of gestures by the total word count.

*Iconic gestures*: In the within-effects model, there were two significant two-way interactions. The first one was between iconic gesture frequency and condition, χ2 (1) = 8.42, *p* = .004; the second one was between iconic gesture frequency and MIT, χ2 (1) = 8.05, *p* = .005. Including iconic gestures, the three-way interaction between iconic gesture frequency, condition and MIT did not improve the model, χ2 (1) = .37, *p* = .542. We conducted simple slope analyses for the two significant two-way interactions. The first two-way interaction between iconic gestures and condition revealed that the use of iconic gestures improved RAT scores only when people were encouraged to gesture, *β* = 2.73, *SE* = 0.63, *p* < .001. The interaction between iconic gestures and MIT revealed that the use of iconic gestures improves RAT scores only for people with mean, *β* = 1.74, *SE* = 0.70, *p* = .01, and below the mean MIT skills, *β* = 3.00, *SE* = 1.30, *p* = .02, but not for those with MIT skills above the mean, *β* = 0.47, *SE* = 0.38, *p* = .22.

When we ran the between-effects model for iconic gestures for GE1 and GE2, we found a significant three-way interaction between iconic gesture frequency, group and MIT, χ2 (1) = 7.89, *p* = .005. As all simple slopes were significant, we split the data into Group 1 and Group 2 and ran the same models, this time only with iconic gesture frequency and MIT as fixed effects, and subject and item as random intercepts. In the model of Group 1, there was a main effect of iconic gestures, χ2 (1) = 27.71, *p* < .001, *β* = 0.77, *SE* = 0.19, *p* < .001. In Group 1’s gesture-encouraged condition, regardless of the MIT skills, the more iconic gestures participants produced, the better they did on the RAT. When we ran the model for Group 2’s gesture-encouraged condition, we found that the two-way interaction between iconic gestures and MIT significantly improved the model, χ2 (1) = 7.50, *p* = .006. The simple slope analyses showed that only for participants with mean, *β* = 0.82, *SE* = 0.30, *p* = .01, and above the mean MIT scores, *β* = 1.42, *SE* = 0.48, *p* < .001, the frequency of iconic gesture use was positively associated with RAT scores. These results show that when people have already been exposed to the task, regardless of their mental imagery skills, they can benefit from iconic gestures, however, if they are exposed to the task for the first time, only those with a certain mental imagery capacity benefit from iconic gestures when solving the RAT task.

The results related to iconic gestures showed that: 1) the positive association between iconic gestures and RAT scores is only true when people are encouraged to gesture and after they have had previous experience with the task; 2) regardless of whether gestures were encouraged or not, for people with average and below the average mental imagery skills, there was a positive association between iconic gestures and RAT performance; 3) when gestures are encouraged and people are faced with the RAT task for the first time, only for those with mental imagery performance that is above the sample’s average, there is a positive association between the RAT and iconic gestures.

An implication of those findings could be that unlike beat gestures, which hamper RAT performance in people with lower mental imagery capacity, iconic gestures can improve their RAT performance when encouraged. Moreover, while beat gestures help those with high mental imagery skills successfully solve the task after they have had practice with it, iconic gestures help those with low mental imagery capacity.

*Metaphoric gestures*: There were not any significant effects in the within-effects model including metaphoric gestures. As it comes to the between-effects model (GE1 and GE2), the three-way interaction between metaphoric gestures, MIT and group significantly improved the model, χ2 (1) = 4.86, *p* = .03. Simple slope analyses showed that for participants with mean MIT skills in Group 1, when encouraged to gesture, there was a negative association between metaphoric gesture frequency and RAT performance, *β* = - 0.21, *SE* = 0.10, *p* =.03. These effects were not significant for those with low, *β* = - 0.26, *SE* = 0.20, *p* =.19, or high MIT, *β* = - 0.16, *SE* = 0.10, *p* =.09. In Group 2’s encouraged condition, however, the relationship between metaphoric gestures and RAT scores was negative and significant, regardless of MIT skills: MIT (-1 *SD*), *β* = - 0.32, *SE* = 0.16, *p* =.05; MIT (*Mean*), *β* = - 0.69, *SE* = 0.14, *p* < .001; MIT (+1 *SD*), *β* = - 1.06, *SE* = 0.24, *p* < .001.

The negative association between metaphoric gestures and the RAT needs further investigation. Most of the triads in the RAT conveyed a literal meaning with few of them having metaphoric connotations, therefore, thinking in metaphoric terms might have hampered successful RAT performance. Moreover, metaphoric gestures were the least frequently used gesture types in the sample, hence, these results might not be sufficient to draw adequate conclusions.

*Deictic gestures:* In the within-effects model including deictic gestures, there was a significant main effect of deictic gesture frequency, χ2 (1) = 10.31, *p* < .001. Regardless of condition or mental imagery ability, deictic gesture frequency was positively associated with RAT performance, *β* = 2.08, *SE* = 0.70, *p* =.003. However, in the between-effects model, the three-way interaction between deictic gestures, group, and mental imagery significantly improved the model, χ2 (1) = 10.31, *p* < .001. There was a significant positive association between deictic gesture frequency and RAT performance regardless of MIT scores or group except for people with MIT skills below the mean in Group 2, *β* = - 0.19, *SE* = 0.13, *p* =.15. These results show that pointing can have a beneficial effect on solving RAT problems, however, if people with lower MIT who have not had previous experience with the task, make deictic gestures when encouraged to gesture, it might not lead to the same positive outcomes.

*Palm-revealing gestures:* In the within-effects model including palm-revealing gestures there were not any significant effects. However, in the between-effects model, including the three-way interaction between palm-revealing gesture frequency, group and mental imagery significantly improved the model fit, χ2 (1) = 4.53, *p* = .03. In both groups, participants with MIT scores below the mean, *β* = - 0.27, *SE* = 0.11, *p* =.02, and those at the mean, *β* = - 0.15, *SE* = 0.06, *p* =.01, showed a negative association between palm-revealing gestures and RAT scores. As palm-revealing gestures are gestures of uncertainty, they convey participants’ hesitation and uncertainty (“I don’t know”, “That’s all.”) about their answers. As this trend is not statistically significant for those with MIT above the mean, this might be indicating that they somehow overcome the negative effects of palm-revealing gestures.
