# Supplementary material for: The creative interplay between hand gestures, convergent thinking, and mental imagery
Source: PLoS One. 2023 Apr 6;18(4):e0283859. doi: 10.1371/journal.pone.0283859 (PMC10079032; doi:10.1371/journal.pone.0283859)
Supplement: Attachment — Submitted filename: Response to Reviewers.docx [file pone.0283859.s002.docx]

**Response to Reviewers**

**Reviewer 1:** The target paper presents and discusses the results of one study aiming to clarify the relationship between hand gestures, mental imagery, and convergent thinking. The study is timely and of relevance for, even though several papers disclose a relation between hand gesturing and divergent thinking, no study to date extended these findings to convergent thinking.

Overall, the manuscript is well written and clear. The state of the art is particularly clear and thorough, but also straightforward in clarifying the hypothesis and its rational. The design of the study is clever (particularly on the manipulation of the variables both within and between subjects), the analyses appropriate and the results clear.

For all the above, I endorse this manuscript to be published. I have but a few minor points and suggestions.

**Response:** We thank the reviewer for their positive feedback and helpful comments on the paper.

**Reviewer 1:** The first sentence in the last paragraph on page 7 (staring on line 195) is a bit awkward to read and tries to squeeze information which would be easier to follow if presented in two or three separate sentences.

**Response:** We have revised the sentence by splitting it into two separate sentences and paraphrasing it as follows: *“There have not been any studies investigating gestures’ effects on convergent thinking. However, research has shown that fluid arm movements, squeezing a ball, or enacting metaphors with hands could improve convergent thinking [1, 2, 46, 47].”*

**Reviewer 1:** First paragraph of the results section (line 415). This information would most naturally fit in the participants section. Also, it goes against what is stated in that section, which reads "Therefore, the data of 80 participants were included in the final analysis". Likewise, shouldn't the abstract read that 78, instead of 80, participants were tested?

**Response:** We have moved the first paragraph of the Results section to the Participants subsection of the Method section (p.12, line 321) explaining the reason for the discrepancy in the two sample sizes. We have also included the descriptive statistics (mean age, standard deviation, and the number of females) of the RAT sample. The newly added paragraph is as follows:

*“For the RAT analyses, two participants were later excluded because one of them was not shown the RAT triads on the screen, but the experimenter only read them out loud; and the second one was excluded because they were an outlier due to previous research familiarity with the task. Thus, our final sample consisted of 78 participants (Mage = 21.3, SD = 2.47; 50 females) for the RAT analyses and 80 participants (Mage = 21.3, SD = 2.46; 52 females) for the vRAT analyses.”*We have also removed the number of the sample size from the abstract, so it is not confusing to the reader:

*“We tested young adults on verbal and visual convergent thinking, controlling for their mental imagery skills.”*

**Reviewer 1:** The figures seemed to me to be of low quality. Also, it would be preferable if the vertical axis specified, in each image, what score is being plotted (as they are, all images just read "score" and the reader has to refer to the caption or the legend). Finally, the labels of the plots (A and B) should be presented in the top left corner of each plot, instead of in the bottom left corner.

**Response:** We have replaced the initial figures with higher-quality ones. We have changed the names of the vertical axes from “Score” to “RAT Score Predicted Probabilities.” Labels (A and B) have been moved to the upper left corners. We have replaced the grey background and grid lines with a white background to make the slopes easier to read. We have changed the acronyms (GS1, GE1, and GE2) in the graphs with the full names of the groups and conditions since the space allows, and again, it makes the graphs easier to read and grasp. Finally, we removed the acronyms from the captions as they are no longer needed.

**Reviewer 2:** This is a very interesting research! Although the efforts to study the dynamics of convergent thinking are known in literature, the present study gives a fresh and comprehensive value in determining associations and potential linkage between gesticulation, mental representation and mental imagery. Further research areas are also properly addressed as the present work is a country located-focused.

**Response:** We thank the reviewer for their thoughtful and encouraging comments.
