# Supplementary material for: The creative interplay between hand gestures, convergent thinking, and mental imagery
Source: PLoS One. 2023 Apr 6;18(4):e0283859. doi: 10.1371/journal.pone.0283859 (PMC10079032; doi:10.1371/journal.pone.0283859)
Supplement: Attachment — Submitted filename: Response to Reviewers.docx [file pone.0283859.s003.docx]

**Response to Reviewers**

**Reviewer 1:** The authors adequately addressed all my comments and the manuscript is considerably improved. In particular, the new figures are much easier to read and more clearly convey the found outcomes.

**Response:** We thank the reviewer for their original comment and also believe that their suggestion has improved the quality of the figures and the paper overall.

**Reviewer 3:** The authors kindly revised the papers according to the comments. Some gesture recognition related papers should be cited.

[1] X. Liu, H. Shi, H. Chen, Z. Yu, X. Li, and G. Zhao. “iMiGUE: An Identity-free Video Dataset for Micro-Gesture Understanding and Emotion Analysis,” IEEE/CVF conference on computer vision and pattern recognition, pp. 10631-10642, 2021.

[2] X. Liu, H. Shi, X. Hong, H. Chen, D. Tao, and G. Zhao “3D Skeletal Gesture Recognition via Hidden States Exploration,” IEEE Transactions on Image Processing, Vol. 29, pp. 4583–4597,2020.

[3] Z. Yu, B. Zhou, J. Wan, P. Wang, H. Chen, X. Liu, S. Li, and G. Zhao. “Searching Multi-Rate and Multi-Modal Temporal Enhanced Networks for Gesture Recognition,” IEEE Transactions on Image Processing, 2021.

[4] X. Liu and G. Zhao, “3D Skeletal Gesture Recognition using Sparse Coding of Time-Warping Invariant Riemannian Trajectories,” IEEE Transactions on Multimedia, Vol. 23, pp. 1841–1854, 2021.

**Response:** We thank the reviewer for their suggested readings. We addressed them in an additional paragraph that we included in the Discussion section (line 808-822) and updated the reference list accordingly (line 1076-1093). As the suggested papers were not very relevant to our claims, we did not go into much detail when discussing them.

The newly added paragraph is as follows: *“Last, one of the main challenges both in creativity assessment and gesture coding can be achieving high interrater reliability, mainly due to subjectivity in human ratings. Although convergent thinking measurement is immune to human bias because correct responses are usually predetermined, classical divergent thinking measures, such as the AUT, are prone to rater subjectivity. These limitations are currently being addressed by automation of creativity assessment with computational methods that use natural language processing [89, 90]. Moreover, newly emerging technological improvements, such as machine-learning algorithms also offer a solution to the challenges in gesture coding (e.g., recently proposed state-of-the-art gesture recognition methods [91-94]). Even though these studies used large datasets to train their models, there is still a big diversity in how people use their gestures, which makes it difficult to train machines to detect and classify gestures. Alternatively, human raters benefit from other verbal and nonverbal cues, such as speech, prosody, and facial expressions, to identify and classify hand gestures. Even if training machines for gesture recognition could be an arduous task, it is a promising path for valuable interdisciplinary work between the fields of psycholinguistics and computer sciences.”*
